# Localization and Tracking of an Indoor Autonomous Vehicle Based on the Phase Difference of Passive UHF RFID Signals

**DOI:** 10.3390/s21093286

**Published:** 2021-05-10

**Authors:** Yunlei Zhang, Xiaolin Gong, Kaihua Liu, Shuai Zhang

**Affiliations:** School of Microelectronics, Tianjin University, Tianjin 300072, China; zyunlei@tju.edu.cn (Y.Z.); liukaihua@tju.edu.cn (K.L.); shuaifirst@tju.edu.cn (S.Z.)

**Keywords:** indoor localization, radio frequency identification (RFID), Kalman filter (KF), semi-definite programming (SDP)

## Abstract

State-of-the-art radio frequency identification (RFID)-based indoor autonomous vehicles localization methods are mostly based on received signal strength indicator (RSSI) measurements. However, the accuracy of these methods is not high enough for real-world scenarios. To overcome this problem, a novel dual-frequency phase difference of arrival (PDOA) ranging-based indoor autonomous vehicle localization and tracking scheme was developed. Firstly, the method gets the distance between the RFID reader and the tag by dual-frequency PDOA ranging. Then, a maximum likelihood estimation and semi-definite programming (SDP)-based localization algorithm is utilized to calculate the position of the autonomous vehicles, which can mitigate the multipath ranging error and obtain a more accurate positioning result. Finally, vehicle traveling information and the position achieved by RFID localization are fused with a Kalman filter (KF). The proposed method can work in a low-density tag deployment environment. Simulation experiment results showed that the proposed vehicle localization and tracking method achieves centimeter-level mean tracking accuracy.

## 1. Introduction

In recent years, a considerable amount of robots have been used in the industrial field. As a result, the positioning of mobile robots has received tremendous attention. However, almost all industrial mobile robots work in indoor environments, where the GPS signal cannot be used for localization. To overcome this limitation, several sensors have been integrated into mobile robots for positioning. Mobile robot positioning schemes can be divided into two categories: relative and absolute [1]. The relative positioning schemes use sensors, such as inertial components and wheel encoders, to obtain the motion vector of the mobile robot. On the other hand, absolute positioning schemes get the position of the robot directly using the indoor position methods. The fusion of these two positioning schemes solves the problem that the relative positioning cannot obtain the initial position. Moreover, the positioning result is more accurate than only using one of these two methods. The most popular fusion algorithms are filter-based methods, including the Kalman filter (KF) and the particle filter (PF).

Ultra-high-frequency (UHF) radio frequency identification (RFID) is a promising technique for indoor localization, which has been widely studied in the past decade. RFID indoor localization is based on the backscatter signal of the RFID tag. The backscatter signal has three dimensions, which are the signal strength, signal phase, and signal frequency. Using these three features of the backscatter signal, the position of RFID tags or reader antennas is obtained by localization algorithms. Localization algorithms are composed of three groups: ranging-based localization algorithms, ranging-free localization algorithms, and SAR-based methods [2]. Ranging-based UHF RFID localization algorithms are made up of the received signal strength indicator (RSSI) [3], the phase difference of arrival (PDOA) [4], and the angle of arrival (AOA) [5]. With reference tags, ranging-free localization algorithms locate tags by online pattern matching [6,7,8]. The SRA-based localization methods move tags or readers on a known trajectory and use measured phases to calculate the coordinates [9].

RFID tags are so inexpensive and easy to deploy that it is appropriate to use RFID localization as the absolute positioning scheme of mobile robot positioning. In this application scenario, the RFID reader is deployed on the mobile robot, and RFID tags are placed in different locations in the indoor environment. The absolute positioning problem of the mobile robot is translated into the positioning of the RFID reader. By querying tags, the RFID reader receives the backscatter signals of tags with known coordinates. The coordinates are stored in the tag chip memory, which is read every query round, or stored on a server used by the localization software directly. The estimate of absolute position is obtained by the ranging-free or ranging-based RFID localization algorithms. However, the estimate of absolute position always has a large error. Fusing the estimate with the observed measurement, which is acquired from the sensors, the error is mitigated.

In this paper, a novel RFID-based indoor mobile robots localization and navigation system is presented. Rather than relying on the RSSI of the RFID backscatter signal to locate the robot, the signal phase was used. Different from existing phase-based methods, the dual-frequency PDOA ranging method was exploited. This method requires deploying much fewer tags in the working environment than landmark-based methods, which require more tags to improve positioning accuracy. In addition, RSSI ranging-based methods also need an intensive tag deployment environment. In actual application scenarios, such as libraries and warehouses, caused by the multipath effect, the received signal strength (RSS) path loss model has a large error, which is known as small-scale fading. With this effect, the RSS varies by several dBm in a short distance, which will cause a large positioning error. Since the PDOA ranging method is also affected by multipath propagation, this paper proposed an algorithm to mitigate this effect.

The main contributions of this paper are summarized as follows.

(1) A novel indoor mobile robot localization and navigation system was proposed. The RFID reader is mounted on the mobile robot and obtains the position of the robot by dual-frequency PDOA ranging. This system needs a much lower tag deployment density, which can reduce the tag reading collision.
(2) To mitigate the multipath-caused ranging error, the localization problem was modeled as an optimization problem and relaxed to an SDP problem, which can be solved efficiently.
(3) In order to improve system accuracy, the odometry information, obtained from wheel encoders, is fused with the RFID localization result. The system is transformed into a linear model, and a novel KF is presented to solve the tracking problem. Different from the nonlinear KF methods, the computational requirements are lower. Hence, the time efficiency of the proposed method is high.

The rest of this paper is organized as follows. The related works are presented and discussed in Section 2. Section 3 introduces the problem formulation, which includes the autonomous vehicle movement model, the autonomous vehicle localization problem, and the UHF RFID channel model. Section 4 proposes the localization and tracking algorithms. Simulation experiments are conducted to verify the performance of proposed methods in Section 5. Section 6 provides the conclusion.

## 2. Related Works

In recent years, many RFID-based indoor robot localization systems have been studied [10]. Early works used RFID tags as landmarks, which requires a dense tag deployment to guarantee the position accuracy. Most of these methods actually do not use any feature of the backscatter signal and just use RFID tags as landmarks. In [11], the mobile robot carried a reader antenna on the bottom, and the position could be estimated through the position data of the tags within the recognition area of the reader. Considering the effect of RFID reading fault, an effective fault-tolerant RFID reader localization approach was studied in [12]. Similarly, some autonomous mobile navigation systems based on high-frequency (HF) passive RFID were studied in [13,14,15]. The relationship between the density of the RFID tag distribution and localization precision was analyzed in [16]. In another work, the tags were used to define the desired trajectory of the robot [17]. Subsequently, the received signal strength (RSS) measurement of the backscatter signal was utilized to estimate the position of mobile robots. Using path loss model, the distance between the tag and reader was obtained. The position was estimated by a probabilistic [18,19] method or a geometric method [20]. Thereafter, sensors, such as ultrasonic sensors [21] and odometry sensors [22], were fused with the RFID localization system to reduce the uncertainties. Filter-based algorithms, such as the Kalman filter (KF) [23,24] and the particle filter (PF), are the most popular fusion methods. In [25,26,27], the phase of the backscatter signals was fused with the odometry information, which was obtained from the encoders on the wheels of the vehicle. Moreover, the phase shift of the backscatter signals was used for robot localization [28,29]. A data-driven fusion estimation method based on the unscented Kalman filter (UKF) was given in [30]. Furthermore, a particle filter can also be used as a fusion method [31]. Different from these two types of famous filtering methods, a new extended finite impulse response (EFIR) filter was proposed for RFID-based mobile robot localization and navigation [32,33]. For indoor robot localization methods, which use UHF RFID tags, the performances of different filters were studied in [34]. What is more, the effect of infrastructure and localization algorithms on position accuracy, in RFID-based robot location systems, was investigated in [35]. In [36], a particle filter was employed to use the phase difference between two steps of the mobile robot for localization and tracking. This method is similar to the landmark-based method and requires a high density of tags. Furthermore, extending the problem to three dimensions, the indoor unmanned aerial vehicles localization and tracking problem was studied in [37,38].

## 3. Problem Formulation

### 3.1. Autonomous Vehicle Movement Model

**Figure 1:**
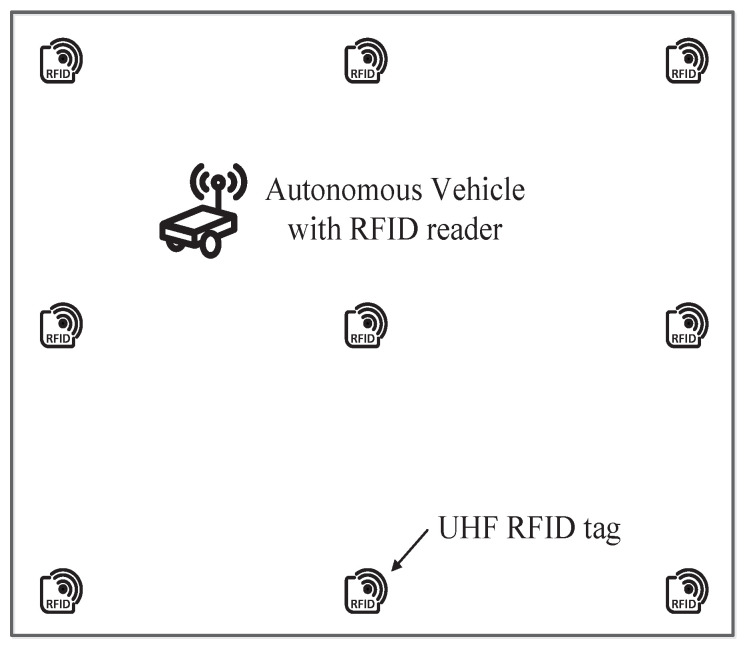
Schematic of the UHF RFID-based indoor autonomous localization and tracking system. The UHF RFID reader is placed on the vehicle, and tags are placed on the floor.

To locate and track the autonomous vehicle in an indoor environment, a UHF RFID reader was deployed on the vehicle, and UHF RFID tags were placed on the floor, as shown in Figure 1. There were *N* tags whose coordinates $t_{i}=\left[x_{i},y_{i}\right],i\in N$ were known. After querying the tags, the reader can obtain the electronic product code (EPC) codes of the tags and extract the phase of the backscatter signal.

**Figure 2:**
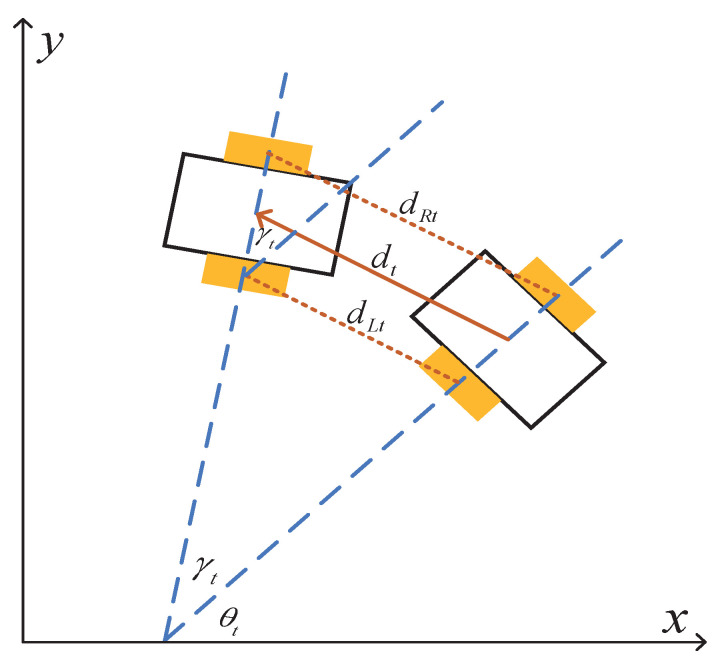
The vehicle state transition of two adjacent points on the trajectory.

The trajectory of the autonomous vehicle was controlled by the left and right wheels. Wheel encoders were installed on each wheel, which recorded the distance traveled by each wheel. This vehicle motion problem was modeled as a state transition model in the paper. Suppose the position of autonomous vehicle at time *t* is $x_{t}=\left[x_{t},y_{t}\right]$ and the orientation is $\theta_{t}$. Let $d_{Lt}$ and $d_{Rt}$ denote the incremental distance of the left and right wheels, respectively. Thus, as shown in Figure 2, the state transition of the vehicle from time *t* to t+1 is given by:

(1) $$x_{t+1}=x_{t}+\frac{d_{t}}{\gamma_{t}}\left[\cos\left(\theta_{t}+\gamma_{t}\right)-\cos\theta_{t}\right],$$

(2) $$y_{t+1}=y_{t}+\frac{d_{t}}{\gamma_{t}}\left[\sin\left(\theta_{t}+\gamma_{t}\right)-\sin\theta_{t}\right],$$

(3) $$\theta_{t+1}=\theta_{t}+\gamma_{t},$$

where $\gamma_{t}$ is the orientation increment from time *t* to t+1 and $d_{t}$ is the vehicle moving distance. These two parameters can be calculated from $d_{Ln}$ and $d_{Rn}$, shown as:

(4) $$d_{t}=\frac{1}{2}\left(d_{Lt}+d_{Rt}\right),$$

(5) $$\gamma_{t}=\frac{1}{l}\left(d_{Rt}-d_{Lt}\right),$$

where *l* is the distance between two wheels. This autonomous vehicle movement model was our original work.

### 3.2. Autonomous Vehicle Localization Problem

The position measurement was obtained from the UHF RFID localization, which was modeled as locating a UHF RFID reader. The phase of the RFID backscatter signal was obtained by a ranging-based localization method. Assume the continuous wave (CW) signal transmitted by the reader is written as:

(6) $$t_{x}\left(t\right)=\sin\left(2\pi ft\right).$$

According to the UHF RFID backscatter channel, the received backscatter signal is:

(7) $$r_{x}\left(t\right)=A\left(t\right)\sin\left(2\pi ft+\varphi \right),$$

where A(t) is the modulation amplitude. φ is the phase delay caused by the signal propagation and can be further written as:

(8) $$\varphi +2k\pi =2\pi \frac{2d}{\lambda }+\varphi_{t}+\varphi_{r}+\varphi_{tag},$$

where $2\pi \frac{2d}{\lambda }$ is the phase delay caused by the signal propagation between the reader and the tag. *d* is the distance between the reader and the tag, and λ is the signal wavelength. *k* is a positive integer, which represents the number of full cycles that the signal propagates within a 2d distance. $\varphi_{t}$, $\varphi_{r}$, and $\varphi_{tag}$ are the phase delay caused by the reader transmit circuits, the tag reflection characteristic, and the reader receiver circuits [39], which are fixed values, independent of the distance between the tag and reader, and can be measured experimentally. Thus, the distance-related phase delay is:

(9) $$\varphi_{d}+2k\pi =\varphi -\varphi_{t}-\varphi_{r}-\varphi_{tag}=\frac{4\pi d}{\lambda }.$$

Since *k* is unknown, it is infeasible to estimate the distance *d* with one frequency band. However, this problem can be solved with the dual-frequency PDOA method. Suppose the two frequencies are $f_{1}$ and $f_{2}$; the phase delay $\varphi_{d1}$ and $\varphi_{d2}$ are:

(10) $$\varphi_{d1}+2k_{1}\pi =\frac{4\pi df_{1}}{c},$$

(11) $$\varphi_{d2}+2k_{2}\pi =\frac{4\pi df_{2}}{c},$$

where *c* is the speed of light. From Equations (10) and (11), the phase difference is written as:

(12) $$\Delta \varphi_{d}=2\Delta k\pi +\frac{4\pi d(f_{1}-f_{2})}{c},$$

where $\Delta k=k_{1}-k_{2}$, $\Delta \varphi =\varphi_{d1}-\varphi_{d2}$. The UHF tags’ readable distance is about 10 to 20 m. Within this distance, the appropriate $f_{1}$ and $f_{2}$ can be selected to make the Δk equal to zero [40]. Consequently, assume that $f_{1}>f_{2}$; when Δφ>0, the distance between the reader and tags can be written as:

(13) $$\hat{d}=\frac{c\Delta \varphi }{4\pi \left(f_{1}-f_{2}\right)}.$$

When Δφ<0, because the range of Δφ is $\left[-\pi ,\pi \right]$, *d* is expressed as:

(14) $$\hat{d}=\frac{c(\Delta \varphi +2\pi )}{4\pi \left(f_{1}-f_{2}\right)}.$$

Due to multipath propagation, the ranging result $\hat{d}$ has an error that approximately obeys a zero mean Gaussian distribution [41]. Therefore, with maximum likelihood estimation, the autonomous vehicle localization problem at time *t* can be modeled as:

(15) $$\begin{array}{c} \min_{x_{t}}\sum_{i=1}^{L}\frac{({\hat{d}}_{i}-\left|\right|x_{t}-t_{i}{\left||\right)}^{2}}{\sigma_{i}^{2}}, \end{array}$$

where ${\hat{d}}_{i}$ is the estimated distance between the vehicle and tag *i*. *L* is the number of readable tags. $x_{t}$ is the coordinate of the autonomous vehicle, denoted as $x_{t}=\left(x_{t},y_{t}\right)$. $\sigma_{i}$ is the standard deviation of the ranging error for ${\hat{d}}_{i}$.

### 3.3. UHF RFID Channel Model

The passive UHF RFID system operates within the ISM band of 902–928 MHz in the USA. The UHF RFID reader communicates with the tags in half-duplex mode, which is known as the backscattering scheme. In this scheme, after sending commands, the RFID reader sends a CW signal, which is used to provide power to the tags. After that, the tag modulates its reflection of the CW signal, which is done by making its antenna work as matched or mismatched.

Based on this scheme, given by [42], the power of the received modulated backscatter signal is:

(16) $$\begin{array}{c} P_{R}=\frac{P_{T}G_{TR}^{2}G_{t}^{2}\lambda^{4}X^{2}M}{{\left(4\pi d\right)}^{4}\Theta^{2}B^{2}F^{2}}, \end{array}$$

where $P_{T}$ is the transmission power. $G_{TR}$ and $G_{t}$ are the load-matched, free-space gain of the reader antenna and tag antenna, respectively. *X* and *M* are the polarization mismatch factor and the modulation factor, respectively. Θ is the on-object gain penalty of tag antenna. *B* and *F* are the losses caused by shadowing and multipath propagation.

**Figure 3:**
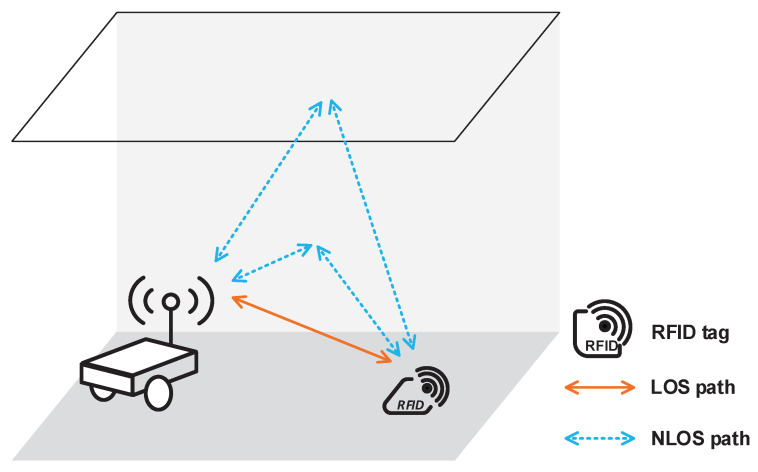
Multipath propagation scenario of the indoor autonomous vehicle navigation system.

Shown in Figure 3, with the reflection from various objects in the indoor environment, signals traverse multiple paths between the RFID tags and the reader. Due to this multipath propagation, a small-scale fading is caused, and the loss factor *F* is written as:

(17) $$F=\frac{{\left|h\right|}^{2}}{d^{2}},$$

(18) h=1de−j2πd−j2πdλλ+∑i=1nΓi1die−j2πdi−j2πdiλλ,

where *h* is the one-way (from reader to tag) channel impulse response. *d* is the distance between the reader and the tag, which also represents the length of the line-of-sight (LOS) path. $d_{i}$ is the length of the *i*-th non-line-of-sight (NLOS) path, which is caused by reflection. *k* is the wavenumber. *n* is the number of NLOS paths. $\Gamma_{i}$ is the small-scale amplitude attenuation of the *i*-th NLOS signal and obeys the Rayleigh distribution. That is because every NLOS path is the sum of many independent rays arriving within the time resolution [43].

**Figure 4:**
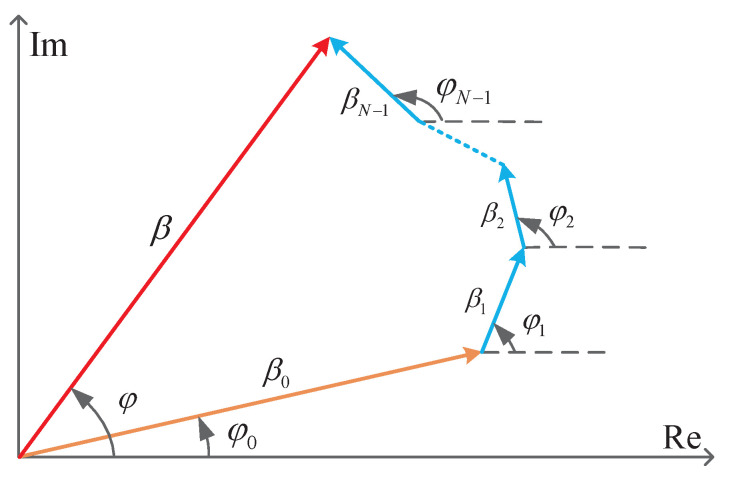
Phase diagram for narrowband signaling propagation on a multipath channel.

Due to the multipath propagation, the transmitted signal arrives at the reader from various directions over a multiplicity of paths. The frequency domain impulse response of the multipath channel is described in Figure 4 where $\beta_{0}$ and $\varphi_{0}$ are the magnitude and phase of the LOS path. $\beta_{i}$ and $\varphi_{i}$ are the magnitude and phase of the NLOS path. Hence, the summed result of the LOS and NLOS path, shown as a vector with a magnitude β and a phase φ, is the final multipath channel impulse response. This vector-summing channel model for UHF RFID system was our original work. Thus, the phase delay of the received signals with respect to the transmission signal is written as:

(19) φ=2arctanImhReh.

## 4. Localization and Tracking Algorithm

In this section, the localization and tracking algorithm is presented. The localization algorithm is based on the dual-frequency PDOA ranging. In theory, the absolute position of the autonomous vehicle can be estimated by using the three coordinates of the tags and the distances from them. The basic position problem is shown in Equation (15), which is usually solved by a least squares method. In this paper, this problem was solved by the convex relaxation method. Assuming the standard deviations are identical for all ranging results, Equation (15) is further written as:

(20) $$\begin{array}{c} \min_{x_{t}}\sum_{i=1}^{L}(d_{i}-\left|\right|x_{t}-t_{i}{\left||\right)}^{2}. \end{array}$$

This problem is a non-convex problem, which can be relaxed to a convex problem with the SDR method, shown as:

(21) $$\begin{array}{cc} \; & \min_{x,b_{i}}\sum_{i=1}^{L}{\left({\hat{d}}_{i}-b_{i}\right)}^{2},\\ s.t.\; & b_{i}\geq \left|\right|x_{t}-t_{i}\left|\right|.\;i=1,2,\ldots L \end{array}$$

Thus, the problem can be solved efficiently by using the convex toolbox [44].

After the mobile vehicle is located, the position coordinate $x_{t}$ is used as the observed measurement. A KF is proposed to fuse this observed measurement position with the state transition position. The original state vector is $x_{t}^{ori}={\left[x_{t}\;y_{t}\;\theta_{t}\right]}^{T}$. The observed measurement vector is $z_{t}={\left[x_{t+1}\;y_{t+1}\right]}^{T}$. The control vector is $u_{t}={\left[d_{t}\;\gamma_{t}\right]}^{T}$. Based on Equations (1)–(3), the state transition equation is:

(22) $$\begin{array}{cc} x_{t+1}= & \left[\begin{array}{c} x_{t+1}\\ y_{t+1}\\ \theta_{t+1} \end{array}\right]\\ = & \left[\begin{array}{c} x_{t}+\frac{d_{t}}{\gamma_{t}}\left(\cos\left(\theta_{t}+\gamma_{t}\right)-\cos\theta_{t}\right)\\ y_{t}+\frac{d_{t}}{\gamma_{t}}\left(\sin\left(\theta_{t}+\gamma_{t}\right)-\sin\theta_{t}\right)\\ \theta_{t}+\gamma_{t} \end{array}\right], \end{array}$$

which is a nonlinear equation. The nonlinear state transition problem is always solved with the EKF or the UKF. In order to solve this problem efficiently, the state transition equation is transformed into a linear equation, shown below.

(23) $$\begin{array}{cc} x_{t+1}= & \left[\begin{array}{c} x_{t+1}\\ y_{t+1}\\ s_{t}\\ c_{t} \end{array}\right]=f\left(x_{t},u_{t}\right)\\ = & \left[\begin{array}{c} x_{t}+\frac{d_{t}}{\gamma_{t}}\left(c_{t}\cos\gamma_{t}-s_{t}\sin\gamma_{t}-c_{t}\right)\\ y_{t}+\frac{d_{t}}{\gamma_{t}}\left(s_{t}\cos\gamma_{t}-c_{t}\sin\gamma_{t}-s_{t}\right)\\ s_{t}\cos\gamma_{t}+c_{t}\sin\gamma_{t}\\ c_{t}\cos\gamma_{t}-s_{t}\sin\gamma_{t} \end{array}\right], \end{array}$$

where $x_{t}={\left[x_{t}\;y_{t}\;s_{t}\;c_{t}\right]}^{T}$, $u_{t}={\left[d_{t}\;\gamma_{t}\right]}^{T}$, $s_{t}=\sin\left(\theta_{t}\right)$, and $c_{t}=\cos\left(\theta_{t}\right)$. Therefore, the update estimate of the KF is given by:

(24) $$\begin{array}{cc} x_{t+1} & =\left[\begin{array}{cccc} 1 & 0 & -\frac{d_{t}}{\gamma_{t}}\sin\gamma_{t} & \frac{d_{t}}{\gamma_{t}}\left(\cos\gamma_{t}-1\right)\;\\ 0 & 1 & \frac{d_{t}}{\gamma_{t}}\left(\cos\gamma_{t}-1\right) & \frac{d_{t}}{\gamma_{t}}\sin\gamma_{t}\\ 0 & 0 & \cos\gamma_{t} & \sin\gamma_{t}\\ 0 & 0 & -\sin\gamma_{t} & \cos\gamma_{t}; \end{array}\right]x_{t}+w_{t}\\ \; & =Fx_{t}+w_{t}. \end{array}$$

The measurement equation is:

(25) $$\begin{array}{cc} z_{t}= & \left[\begin{array}{c} x_{t+1}\\ y_{t+1} \end{array}\right]+v_{t}=\left[\begin{array}{cc} 1 & 0\\ 0 & 1 \end{array}\right]x_{t+1}+v_{t}\\ = & Hx_{t+1}+v_{t}. \end{array}$$

The flow of the localization and tracking algorithm is shown in Algorithm 1. Firstly, the original state vector and the covariance are loaded. After that, according to Equation (24), the predicted state is calculated. The predicted covariance is updated as the same time. Then, using the predicted covariance and the observation covariance, the Kalman gain is obtained. Finally, the updated state is calculated, and the estimated covariance is updated.
**Algorithm 1** UHF RFID-based indoor autonomous vehicle localization and tracking algorithm.**Input:**       {$x_{i}$}: Tags’ coordinates       {$d_{i}$}: PDOA ranging results       $x_{ori}$: Original state vector       $P_{ori}$: Original covariance matrix       {$Q_{t}$}: The covariance of the process noise       {$R_{t}$}: The covariance of the observation noise**Output:** the autonomous vehicle position x1:**while** The autonomous vehicle is on the trajectory **do**2:      **if** t=1 **then**3:         $P_{t|t}=P_{ori}$4:         $x_{t|t}=x_{ori}$5:      **end if**6:      Calculate the predicted state $x_{t|t-1}$ with $x_{t|t-1}=Fx_{t-1|t-1}$7:      Calculate the predicted covariance $P_{t|t-1}$ with $P_{t|t-1}=FP_{t-1|t-1}F+Q_{t}$8:      Calculate the Kalman gain $K_{t}$ with $K_{t}=P_{t|t-1}H{\left(HP_{t|t-1}H^{T}+R_{t}\right)}^{-1}$9:      Use Equation (25) to calculate the measurement $z_{t}$10:    Calculate the updated state $x_{t|t}$ with $x_{t|t}=x_{t|t-1}+K_{t}\left(z_{t}-Hx_{t|t-1}\right)$11:    Calculate the updated estimate covariance $P_{t|t}$ with $P_{t|t}=P_{t|t-1}-K_{t}HP_{t|t-1}$12:    t=t+113:**end while**

## 5. Numerical Results

### 5.1. Simulation Configuration

To evaluate the proposed localization and tracking system, simulation experiments were designed and carried out in MATLAB 2016a. The computer used was a Lenovo desktop equipped with Intel i5 6500 CPU at 3.2 GHz and 4GB DDR3 RAM, and the operating system was Windows 7 64-bit. The experiment place was a two-dimensional square area whose size was 5 m × 5 m. The UHF RFID tags were placed in this area with a square grid layout. The distance between two adjacent tags was 0.5 m. Two frequencies were utilized to read the tags, which were 920 MHz and 925 MHz. The wireless channel impulse response was generated by the channel model proposed in this paper. The phase delay was calculated based on this multipath channel impulse response. The measurement error of the odometer sensor was assumed to be a uniform distribution parameter. The localization error is calculated as:

(26) $$err_{lo}=∥x_{true}-x_{lo}∥,$$

where $x_{true}$ is the ground truth coordinate and $x_{lo}$ is the result of the localization algorithm. The tracking error is calculated as:

(27) $$err_{tr}=∥x_{true}-x_{tr}∥,$$

where $x_{tr}$ is the estimated coordinate of the tracking algorithm. The root mean squared error (RMSE) of the localization error and tracking error is defined as:

(28) $$RMSE_{lo}={\left(\frac{1}{M}\sum_{i=1}^{M}{err_{lo}^{i}}^{2}\right)}^{\frac{1}{2}},$$

(29) $$RMSE_{tr}={\left(\frac{1}{M}\sum_{i=1}^{M}{err_{tr}^{i}}^{2}\right)}^{\frac{1}{2}},$$

where $err_{lo}^{i}$ and $err_{tr}^{i}$ are the *i*-th localization and tracking error, respectively. *M* is the number of steps.

### 5.2. Performance of Tracking with Different Trajectories

**Figure 5:**
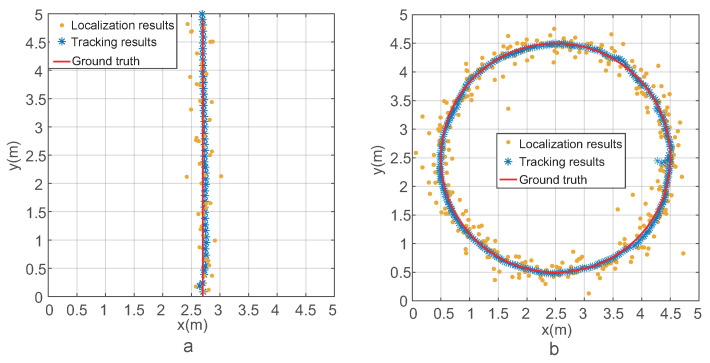
The localization and tracking results when RFID reader could read four neighboring tag. (**a**) The localization and tracking results of the line trajectory. (**b**) The localization and tracking results of the circular trajectory.

To test the tracking algorithm, two trajectories were designed. As shown in Figure 5a,b, one was a straight line, and the other was a circle. The red lines in these two sub-figures are the ground truth of the trajectories. The yellow point is the localization result of the PDOA RFID localization algorithm. Furthermore, the blue asterisks are the results of the tracking algorithm at each step. The movement step was set to be 0.1 m in the straight line trajectory and 0.035 m in the circle trajectory. In these two experiments, it was assumed that the reader could read four neighboring tags. Thus, the localization result was calculated by using the four ranging results. It can be seen that the RFID localization results had obvious errors. For the straight line trajectory and the circle trajectory, the mean localization errors were 0.201 m and 0.196 m. The RFID localization error was caused by the ranging error, which was caused by the multipath propagation of the wireless signal and phase noise. Using the proposed Kalman filter-based tracking algorithm, the tracking errors were 0.116 m and 0.053 m for the straight line trajectory and the circle trajectory, which were 42.3% and 73.0% lower than the localization errors. The reason why the tracking error of the the straight line trajectory was bigger than the circle trajectory was that the movement step of the former was bigger than the latter. It can be seen from Figure 5 that the tracking results, which are blue asterisks, had sufficient accuracy for robot tracking in a warehouse.

### 5.3. Performance of Tracking with Different Numbers of Tags

**Figure 6:**
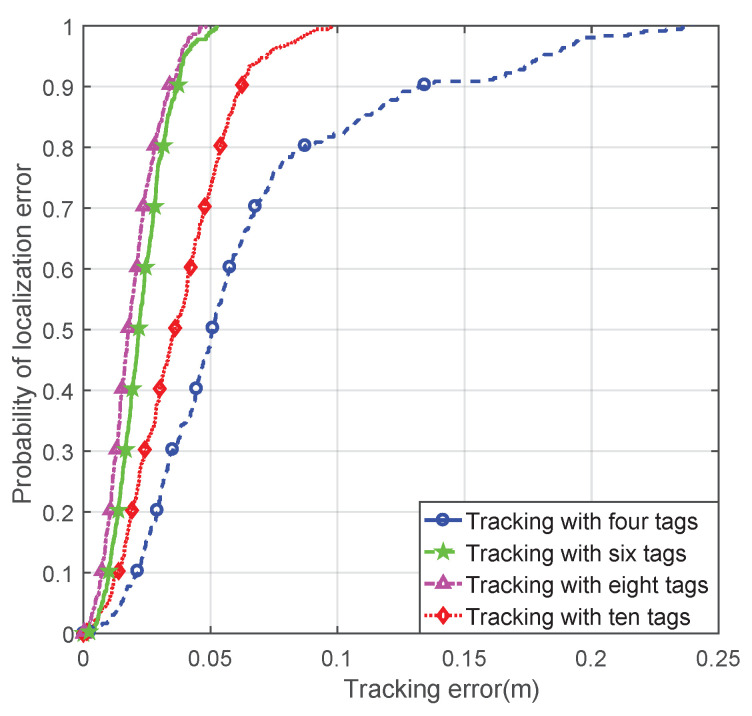
The CDF curves of the circle trajectory tracking error with different numbers of tags.

Another experiment was performed to find the relationship between the number of tags used for localization and the accuracy of the tracking. The straight and circular trajectories were both utilized in this experiment. The experimental environment setting was identical to the previous experiment. The only parameter that was different was the number of tags used for localization, which was set to be four, six, eight, and ten in the experiment. The mean error and RMSE of localization and tracking with different numbers of tags are shown in Table 1. The mean localization errors for these two trajectories using different numbers of tags were similar, which proved the robustness of the proposed channel model and localization algorithm. The cumulative distribution function (CDF) curves of the circle trajectory tracking error with different numbers of tags are shown in Figure 6. The 80% probability tracking error of four, six, eight, and ten tags was 0.026 m, 0.029 m, 0.053 m, and 0.088 m, respectively. It can be seen from Table 1 and Figure 6 that when the number of tags changed from four to eight, the localization and tracking errors reduced. However, when the number of tags was ten, the error increased. This was caused by the increasing localization error. When ten tags were used for localization, the tags at a long distance caused a big ranging error, which made the localization error increase.

### 5.4. Comparison With an RSSI Ranging Method

**Figure 7:**
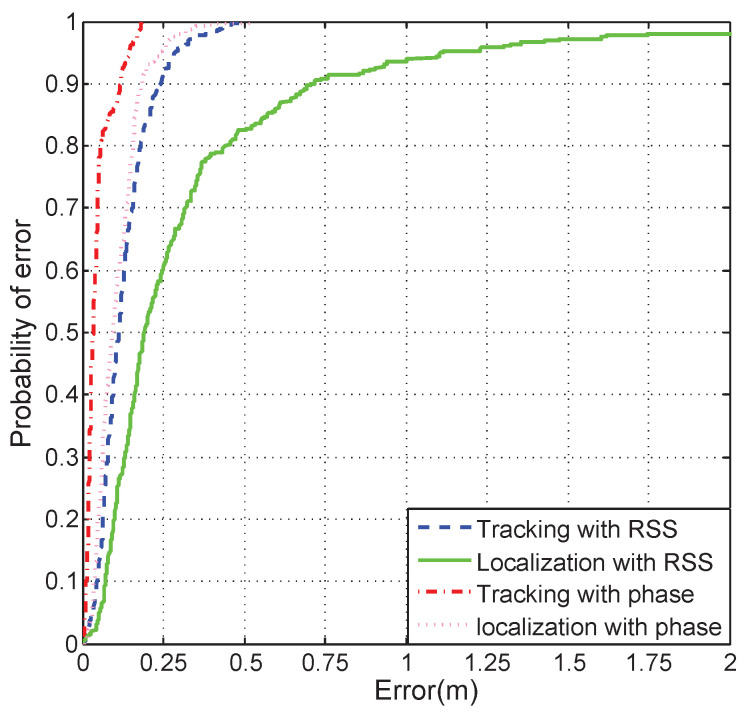
The CDF curves of the circle trajectory tracking and localization error with the RSS and phase.

In order to compare the proposed method with previous works, we designed an experiment to evaluate the localization accuracy and tracking accuracy. Although there are some phase-based autonomous vehicle tracking methods, they require a high-density tag deployment environment to work. Hence, the proposed method was compared with the well-known RSSI ranging method [41], where the RSS of the backscatter signal is employed to estimate the distance between the tag and reader. In this experiment, the autonomous vehicle was moving on the circular trajectory. The number of tags that were utilized to estimate the position of the autonomous vehicle was four. The localization error and tracking error were calculated with Equations (26) and (27). The CDF curves of tracking and localization error with RSS and phase are shown in Figure 7. The 80% probability RSSI-based localization error, RSSI-based tracking error, phase-based localization error, and phase-based tracking error were 0.42 m, 0.18 m, 0.16 m, and 0.088 m, respectively. It can be seen that the error of the RSSI-based localization is much higher than the phase-based localization. Hence, the track accuracy of the proposed phase-based method was higher than the traditional RSSI-based method. This was caused by the RSSI-based ranging method having a poor performance in the multipath environment. The small-scale fading made the ranging result have a big error. Even though the multipath signal propagation also had impacts on the signal phase, the proposed PDOA-based tracking method had a higher accuracy.

## 6. Conclusions

In this paper, the UHF RFID-based indoor autonomous vehicle localization and tracking problem was studied. Different from the previous solutions, the PDOA-based ranging method was employed to calculate the distance between the tags and the autonomous vehicle, which can reduce the density of RFID tags. The UHF RFID localization problem was modeled as an optimization problem whose result was provided as a measurement value to the Kalman filter, whose state transition was based on the odometer sensor measurement. Fusing the RFID localization result and the odometer sensor measurement, the accumulative error problem of the odometer sensor was solved. Simulation experiments were designed and performed to verify the proposed localization and tracking algorithm. The results showed that the tracking accuracy was centimeter level, which is reasonable for most indoor robot tracking applications. Furthermore, comparing with the traditional RSSI-based solutions, the proposed method achieved high localization and tracking accuracy. In the future, the tracking methods for more autonomous vehicle movement models, such as four-wheel vehicles and tracked vehicles, need to be investigated.

## Figures and Tables

**Table 1:** Ranging and localization mean error.

Experiment Setup	Mean Error (m)		RMSE (m)	
	Straight Line	Circle	Straight Line	Circle
Localization with four tags	0.201	0.196	0.221	0.229
Tracking with four tags	0.116	0.053	0.107	0.056
Localization with six tags	0.158	0.163	0.172	0.181
Tracking with six tags	0.075	0.032	0.072	0.028
Localization with eight tags	0.142	0.137	0.167	0.152
Tracking with eight tags	0.06	0.018	0.069	0.023
Localization with ten tags	0.148	0.144	0.175	0.170
Tracking with ten tags	0.087	0.02	0.093	0.032

## Data Availability

Not Applicable.
